# Life history theory and breast cancer risk: methodological and theoretical challenges: Response to “Is estrogen receptor negative breast cancer risk associated with a fast life history strategy?”

**DOI:** 10.1093/emph/eov035

**Published:** 2016-05-21

**Authors:** Athena Aktipis

**Affiliations:** ^1^Department of Psychology, Center for Evolution and Medicine, Center for Social Dynamics and Complexity, Arizona State University, Tempe, AZ, USA

**Keywords:** evolutionary mismatch, tradeoffs, life history theory, cancer susceptibility

## Abstract

In a meta-analysis published by myself and co-authors, we report differences in the life history risk factors for estrogen receptor negative (ER−) and estrogen receptor positive (ER+) breast cancers. Our meta-analysis did not find the association of ER− breast cancer risk with fast life history characteristics that Hidaka and Boddy suggest in their response to our article. There are a number of possible explanations for the differences between their conclusions and the conclusions we drew from our meta-analysis, including limitations of our meta-analysis and methodological challenges in measuring and categorizing estrogen receptor status. These challenges, along with the association of ER+ breast cancer with slow life history characteristics, may make it challenging to find a clear signal of ER− breast cancer with fast life history characteristics, even if that relationship does exist. The contradictory results regarding breast cancer risk and life history characteristics illustrate a more general challenge in evolutionary medicine: often different sub-theories in evolutionary biology make contradictory predictions about disease risk. In this case, life history models predict that breast cancer risk should increase with faster life history characteristics, while the evolutionary mismatch hypothesis predicts that breast cancer risk should increase with delayed reproduction. Whether life history tradeoffs contribute to ER− breast cancer is still an open question, but current models and several lines of evidence suggest that it is a possibility.

Hidaka and Boddy [1] report evidence consistent with the hypothesis that ER− breast cancer risk is associated with a fast life history strategy. In a meta-analysis published by myself and co-authors in this journal, we reported differences in the life history risk factors for estrogen receptor negative (ER−) and estrogen receptor positive (ER+) breast cancers. We did not find an association of ER− breast cancer risk with fast life history characteristics. However, Hidaka and Boddy’s proposal is based on other evidence not included in our meta-analysis, such as differences in socioeconomic status, nutrition, and genetic variants that are associated with fertility. There are a number of possible explanations for the differences between their conclusions and the conclusions we drew from our meta-analysis, including limitations of our meta-analysis and methodological challenges in measuring and categorizing estrogen receptor status.

The first possibility is that ER− breast cancer is not, in fact, associated with fast life history characteristics, despite Hidaka and Boddy’s review of literature that supports this association. Although our meta-analysis showed no association of ER− breast cancer risk with either parity or age of first birth, results of studies included were not consistent, with some finding that ER- breast cancer was in fact associated with higher parity and others finding the opposite. ER− breast cancer is much less common than ER+ cancer, limiting the statistical power of many of these studies. The relationship between ER− status and life history characteristics may be more complex than can be understood with meta-analyses on existing studies with their own limitations.

The second potential explanation for the differences between our results is methodological challenges in breast cancer categorization. Our meta-analysis did not detect a relationship between ER− breast cancer and life history characteristics; this may be because this relationship does not exist or because of inconsistent categorization of breast cancer into ER+ and ER- subtypes. ER status is typically decided based on the cutoff that at least 10% of cells in the biopsy stain positive for estrogen receptors, but this is not entirely consistent across studies, and some recommendations have set the threshold for ER+ as low as 1% [2]. Further, staining of receptors is accomplished using immunohistochemistry methods, which are subject to a variety of methodological challenges including variations in tissue preparation, delay in exposure and length of exposure to antibodies [3, 4]. A study of 150 laboratories in the UK found that many local laboratories classified tumors as having lower ER expression than the UK central laboratory’s scoring system [4], suggesting that tumors with relatively high ER expression may be miscategorized as ER−. This means it may be difficult to find a relationship between ER− breast cancer risk and fast life history characteristics even if such a relationship exists. Given our meta-analysis results which show an association of ER+ breast cancer risk with slow life history characteristics, ER + tumor that are categorized as ER- tumors might introduce errors that limit our capacity to detect an association of ER- breast cancer with fast life history characteristics.

In addition, the ER− breast cancer category is a combination of HER2+ cancers and triple negative (estrogen receptor negative, progesterone receptor negative and HER2 negative) cancers. These tumor types are qualitatively different, have different survival outcomes [5] and may have different life history factor associations. So, even if the ER classification were accurate, we might expect mixed results from the ER− tumors.

Another methodological challenge in categorizing breast cancer receptor status arises from tumor heterogeneity: when only a single biopsy is taken sampling error can lead some breast cancers that may have a large proportion of ER+ cells to be categorized as ER−. If a large number of breast cancers are estrogen sensitive, but are categorized as ER− or vice versa, this limits our ability to detect an association between ER− breast cancer and fast life history strategy. Sampling from multiple regions of the tumor could help to address this limitation, as could measuring the proportion of ER+ cells and using that proportion in analyses of risk factors (rather than binary categories of ER+/−).

Theoretical work completed after this meta-analysis was published suggests that cancer risk should be associated with fast life history characteristics including faster growth, early reproduction and higher fertility [6]. This theoretical result is not consistent with our meta-analysis, with the exception of early menarche, which we found to be associated with both ER+ and ER− breast cancer risk. We also found in our meta-analysis that ER+ breast cancer was associated with slow life history characteristics, which is likely due to evolutionary mismatch between our modern environment and the environment in which our ancestors evolved. This is also not consistent with the predictions of this recently published model. The evolutionary mismatch account posits that increased cancer risk is associated with estrogen exposure due to modern females having 3–4 times more ovulatory cycles than ancestral females. In the absence of the effects due to evolutionary mismatch, the predicted pattern of cancer susceptibility being associated with fast life history strategy [6] might emerge for breast cancer.

Whether life history tradeoffs contribute to ER− breast cancer is still an open question, but current models and several lines of evidence suggest that it is a possibility. Future work using consistent methods for assessing ER−/ER+ status, stratification of ER− cancers into HER2+ and triple negatives, analyzing multiple biopsies and conducting analyses using the proportion of cells staining positive for ER could help resolve these open questions. Other important open questions remain regarding the potential mechanisms that may underlie associations between life history characteristics and breast cancer risk, such as alterations in methylation of tumor suppression genes such as *BRCA1* [7], and the association of fetal microchimerism with differences between subtypes of breast cancer risk [8, 9].

Funding: Application of Evolutionary Principles to Maintain Cancer Control PQ21 NIH/NCI R01CA170595; Genomic and Microenvironemntal Diversity as Drivers of Metastasis in DCIS PQC3 NIH/NCI, R01; 3. Breakthrough Award DoD/Breast Cancer Research Program Genomic and Microenvironemntal Diversity as Drivers of Progression in DCIS.
